# Improving the Accuracy of the Hyperspectral Model for Apple Canopy Water Content Prediction using the Equidistant Sampling Method

**DOI:** 10.1038/s41598-017-11545-x

**Published:** 2017-09-11

**Authors:** Huan-San Zhao, Xi-Cun Zhu, Cheng Li, Yu Wei, Geng-Xing Zhao, Yuan-Mao Jiang

**Affiliations:** 1 College of Resources and Environment, Shandong Agricultural University, Tai’an, 271018 China; 2 Key Laboratory of Agricultural Ecology and Environment, Shandong Agricultural University, Tai’an, 271018 China; 3 College of Horticulture Science and Engineering, Shandong Agricultural University, Tai’an, China, 271018 China

## Abstract

The influence of the equidistant sampling method was explored in a hyperspectral model for the accurate prediction of the water content of apple tree canopy. The relationship between spectral reflectance and water content was explored using the sample partition methods of equidistant sampling and random sampling, and a stepwise regression model of the apple canopy water content was established. The results showed that the random sampling model was Y = 0.4797 − 721787.3883 × Z_3_ − 766567.1103 × Z_5_ − 771392.9030 × Z_6_; the equidistant sampling model was Y = 0.4613 − 480610.4213 × Z_2_ − 552189.0450 × Z_5_ − 1006181.8358 × Z_6_. After verification, the equidistant sampling method was verified to offer a superior prediction ability. The calibration set coefficient of determination of 0.6599 and validation set coefficient of determination of 0.8221 were higher than that of the random sampling model by 9.20% and 10.90%, respectively. The root mean square error (RMSE) of 0.0365 and relative error (RE) of 0.0626 were lower than that of the random sampling model by 17.23% and 17.09%, respectively. Dividing the calibration set and validation set by the equidistant sampling method can improve the prediction accuracy of the hyperspectral model of apple canopy water content.

## Introduction

As an important index of plant growth, the water content can be used to detect the degree of water deficit and ultimately to guide rational irrigation^1^. The traditional chemical analysis method for assessing leaf water content utilizes destructive sampling. While the precision is high, the high consumption, burdensome complexity, and difficult storage and transportation of field samples make this method unable to meet the requirement of rapid on-site nondestructive detection. As a type of real-time, fast and non-destructive technology, hyperspectral technology is playing an increasingly important role in the detection of crop growth and acquisition of nutrients^2–4^ and has become an active research topic in agricultural remote sensing^5^. Spectral techniques have been used to predict the water content of grain crops and fruit trees^4–7^. Since the 20th century, Penuelas *et al*.^8–11^ have studied the effects of drought on crop spectral characteristics and the spectral response to leaf water deficits. A model for the diagnosis of leaf water content using the absorption characteristics of 1450 nm and 1650–1850 nm band spectra has been proposed.

Subsequently, scholars have found that because of the strong absorption characteristics of water at 1450 nm and 1950 nm, 2 main reflection peaks are formed in the vicinity of 1650 nm and 2200 nm, and the water absorption rate is obtained using the strong absorption characteristics of the band^12, 13^. Based on this technique, near the beginning of the 21^st^ century, the use of spectral technology to predict vegetation water content was in its most active period. Scholars began to explore different methods of plant water content inversion. Ceccato *et al*.^14^ used the ratio of the short-wave infrared band (700–1300 nm) to the near-infrared band (1600 nm and 820 nm) to predict the vegetation water content and found that a combination of SWIR and NIR (only influenced by these two parameters) offered a higher accuracy than the single-band model for water content prediction. Xicun Zhu *et al*.^15^ established a prediction model of apple leaf water content based on the spectral index and found that the sensitive bands of water content in apple leaves were concentrated primarily in the near-infrared region. Principal component regression analysis has been used to establish a prediction model of the water content of apple leaves and offers satisfactory sensitivity and stability. Yang Yong *et al*.^16^ used stepwise regression analysis to develop a citrus leaf water content model.

When the water content regression model is constructed, the calibration set and the validation set of the partition model are often sampled randomly^17, 18^. The advantage of this method is that the operation is simple, but it is easy to cause polarization of the system, thus affecting the accuracy of the model and test. The calibration set and the validation set of the equidistant sampling method can make the extracted samples uniformly distributed on the whole, which can eliminate the polarization caused by the simple random sampling^19^. At present, studies exploring the interaction impact capacity between sensitive wavelengths are insufficient, particularly in the areas of modeling and test samples. Moreover, these studies typically use the random sampling method, and the use of the equidistant sampling method is rare.

This study explores the relationship between reflectance spectroscopy and the water content of an apple canopy in the full bearing period. A wavelength sensitive to apple canopy water content is selected. Then, the cross variables are identified, and a stepwise regression model of the water content is obtained. Subsequently, the accuracy of the model built is compared using the methods of equidistant sampling and simple random sampling, and the equidistant sampling method is verified based on the accuracy of the model to provide a theoretical basis for water monitoring and reasonable timely irrigation of apple trees.

## Results and Analysis

### Spectral reflectance features of apple canopies

**Figure 1:**
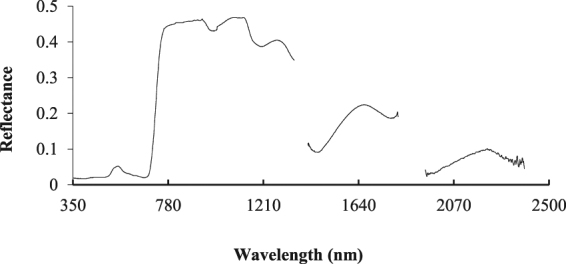
Hyperspectral curves of the original reflectance of an apple canopy. The spectral reflectance of 90 groups of pretreated samples was characterized by the average. The original spectral reflectance curve of the apple canopy was obtained.

Studies have shown that there is a close relationship between plant water content and its canopy reflectance^1, 13, 14^. So we used ASD FieldSpec4 to obtain the DN value of apple canopy, and then converted it into reflectance through a specific mathematical transformation. The spectral reflectance of 90 groups of pretreated samples was characterized by the average, and the original spectral reflectance curve of the apple canopy was obtained, as shown in Fig. 1. The curve shows peaks near 546 nm, 760 nm, 1170 nm, 1660 nm, and 2220 nm and troughs near 667 nm, 981 nm, 1196 nm, 1456 nm, 1780 nm, and 1950 nm. The “Green Peak” appears at 546 nm, and the “Red Valley” appears at 667 nm. The troughs at 981 nm 1196 nm, 1456 nm, 1780 nm, 1950 nm and approximately 2220 nm are produced by the strong absorption characteristics of water content in the leaves.

### Correlation analysis between the original spectral reflectance and the water content of the apple canopy

**Figure 2:**
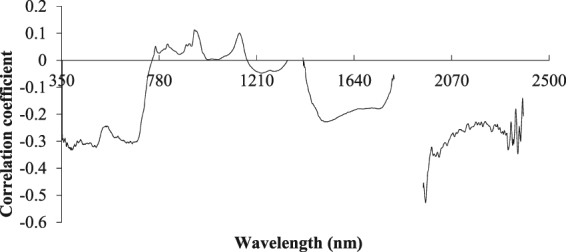
Correlation image between the original reflectance of the apple canopy and its water content. The curve was obtained by correlation analysis between the original spectrum and its water content. Positive and negative correlations are minimal.

The correlation between the original spectral reflectance and the water content of the apple canopy was analyzed, and the results are shown in Fig. 2. After smoothing and error elimination, substantial noise remains in the mid-infrared band of 1950–2380 nm, which is due to the strong absorption of water vapor in the atmosphere when the canopy reflectance was measured. It is challenging to separate the contribution of atmospheric water vapor and vegetation water in the spectral reflectance. At present, progress in this area cannot meet the quantitative remote sensing requirements of vegetation water content under field conditions; thus, the elimination process was conducted on the band. The maximum correlation coefficient of 0.3233 appeared at 380 nm. The data were not suitable for modeling.

### Correlation analysis between the logarithm of the spectral reflectance and its first derivative with the water content of apple canopy

**Figure 3:**
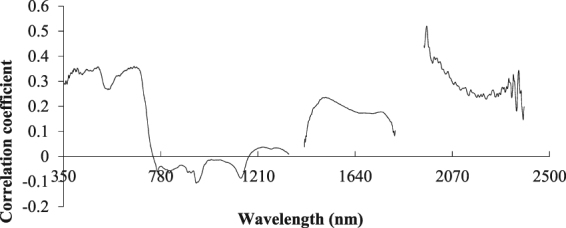
Correlation analysis of the logarithm of the reciprocal of the canopy spectral reflectance and the water content in the apple canopy. The reciprocal of the logarithm transformation was used to obtain the correlation curve.

The inversion of the logarithm of the original spectral reflectance data has been widely used in the field of spectroscopy. The results showed that the spectral reflectance data tended to enhance the spectral differences of visible light and tended to weaken the multiplicative factors caused by the change of illumination conditions after reciprocal logarithmic transformation. The derivative spectrum can eliminate the baseline drift, strengthen the spectral band characteristics and overcome the overlap of spectral bands. This approach is also a common method of spectral data processing. To improve the correlation between the spectral reflectance and water content, the mathematical transformation of the logarithm of the original spectral reflectance was made, and the first derivative transformation was performed on this basis. Figure 3 shows the correlation analysis between the logarithm of the reciprocal of the apple canopy reflectance and the water content. The correlation between the original spectral reflectance of apple tree canopy and the water content was improved after taking the logarithm of the reciprocal, but the change was not substantial. The trend of the peak valley position and correlation curve was essentially the same as that in Fig. 2, and the correlation was still low. The maximum correlation coefficient was 0.3570 at 660 nm, which was not suitable for the establishment of the model.

**Figure 4:**
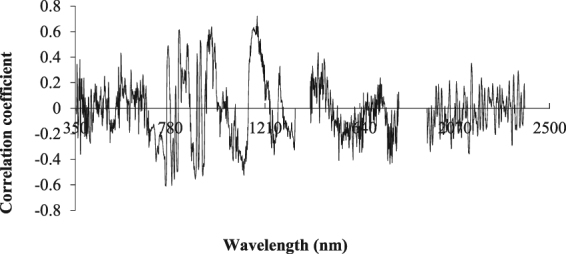
Correlation image between the first derivative of the logarithm of the reciprocal of the original reflectance and the water content. The curve was obtained by the first derivative transformation of the logarithm of the reciprocal.

Figure 4 shows the correlation analysis between the first derivative and the water content based on the reciprocal logarithm. The correlation between the apple canopy reflectance and the water content increased significantly after the transformation, and the most easily detectable band was the near-infrared band. The correlation coefficients of R_760_, R_821_, R_893_, R_967_, R_1114_, and R_1174_ were approximately 0.6. This finding proved that the response of the soil spectrum could be transformed into a constant by the first-order derivative, allowing stabilization.

### Construction of cross variables

Sometimes, the partial effect, the elasticity, or the semi elasticity of a dependent variable is naturally dependent on the numerical size of the other explanatory variable.

A cross variable (interaction term) characterizes the effect of one variable on the ability of another variable to affect the dependent variable. This approach can be used to investigate the cross effect between the core explanatory variables and the control variables using the cross variable prediction model. When constructing the independent variable of the model, the application of the cross variable is relatively extensive, and the cross variables can investigate the cross effect between the core explanatory variable and a control variable^20, 21^. This study focuses on the interactive influence ability of the wavelength near the peaks and valleys, the wavelength of the vegetation red edge position and wavelength on the smooth curve; thus, we selected the wavelength of R_967_ and R_1174_ with high correlation that was close to the peak valley location, the wavelength of R_760_ near the vegetation red edge position and the wavelength of R_821_ on the smooth curve. The cross variables were constructed as Z_1_ = R_760_ × R_821_, Z_2_ = R_760_ × R_967_, Z_3_ = R_821_ × R_967_, Z_4_ = R_821_ × R_1174_, Z_5_ = R_967_ × R_1174_, and Z_6_ = R_760_ × R_1174_. The significance of the cross variables were tested to determine the number of variables at the significant level when the stepwise regression model was established.

### Construction and optimization of the apple canopy water content inversion model

The stepwise regression model of the water content of the apple canopy was established using Z_1_, Z_2_, Z_3_, Z_4_, and Z_6_ as the independent variables of the constructed variables.

### Inversion model and test of the water content in the apple canopy based on simple random sampling

The data was input into the DPS data processing software. The significant level of P > 0.05 was successively eliminated for the independent variables Z_2_, Z_1_, and Z_4_ at the time the significant level P of the variance analysis of equation F was less than 0.05, and there was no residual autocorrelation. The selected independent variables were Z_3_, Z_5_, and Z_6_ (P < 0.05), of which Z_3_ and Z_6_ reached an extremely significant level (P < 0.01). According to Table 1, the following model was established using the data of 72 sets of the calibration set as the dependent variable:

$$Y=0.4797\,-\,721787.3883\times {Z}_{3}\,-\,766567.1103\times {Z}_{5}\,-\,771392.9030\times {Z}_{6}$$

**Figure 5:**
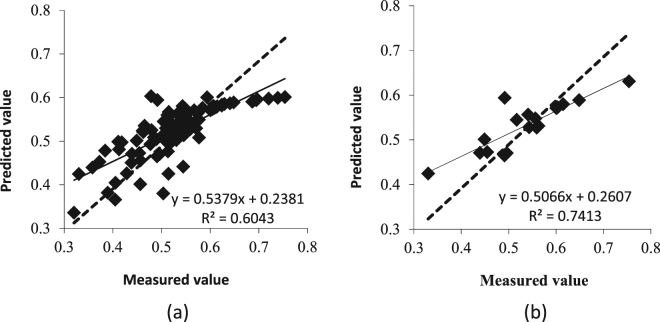
Estimation accuracy test of the cross-variable model based on simple random sampling. (**a**) Test of calibration set. (**b**) Test of validation set. The determination coefficient of the equation indicates the accuracy of the model. The larger the coefficient of determination, the higher the accuracy of the model.

A simple random sampling method was used to extract 18 sets of data (Table 1). The prediction effect of the model on the calibration set and validation set is shown in Fig. 5.

**Table 1:** The water content statistics of apple trees from the cross-variable model based on simple random inspection.

Sample	N	Max (%)	Min (%)	Mean (%)	Std Dev	CV (%)
Total	90	75.3	32.0	52.5	0.087	16.6
Calibration	72	73.9	32.0	52.6	0.086	16.3
Validation	18	75.3	33.0	53.6	0.091	16.9

Note: N: number of samples; Std Dev: standard deviation; and CV: coefficient of variation.

### Inversion model and test of the water content in the apple canopy based on equidistant sampling

The data was input into the DPS data processing software. The significant level of P > 0.05 was successively eliminated for the independent variables Z3, Z1, and Z4 at the time the significant level P of the variance analysis of equation F was less than 0.05, and there was no residual autocorrelation. The selected independent variables were variables were Z_2_, Z_5_, and Z_6_ (P < 0.05), of which Z_2_ and Z_6_ reached an extremely significant level (P < 0.01). According to Table 2, the following model was established using the data of 72 sets of the calibration set as the dependent variable:

$${\rm{Y}}=0.4613\,-\,480610.4213\times {Z}_{2}\,-\,552189.0450\times {Z}_{5}-1006181.8358\times {Z}_{6}$$

**Figure 6:**
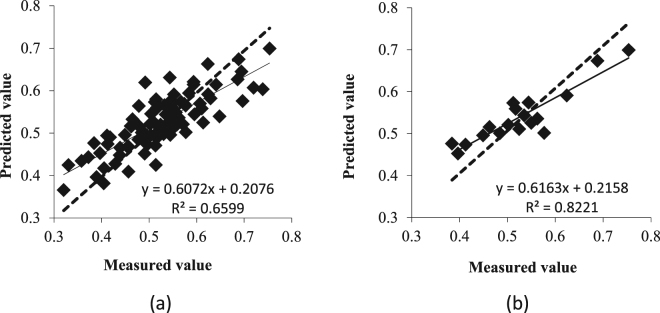
Estimation accuracy test of the cross-variable model based on equidistant sampling. (**a**) Test of the calibration set. (**b**) Test of the validation set. The determination coefficient of the equation indicates the accuracy of the model. The larger the coefficient of determination, the higher the accuracy of the model.

An equidistant sampling method was used to extract 18 sets of data (Table 2). The prediction effect of the model on the calibration set and validation set is shown in Fig. 6.

**Table 2:** The water content statistics of apple trees from the cross-variable model based on systematic sampling.

Sample	N	Max (%)	Min (%)	Mean (%)	Std Dev	CV (%)
Total	90	75.3	32.0	52.5	0.087	16.6
Calibration	72	73.9	32.0	52.6	0.086	16.3
Validation	18	75.3	38.4	52.7	0.093	17.6

Note: N: number of samples; Std Dev: standard deviation; and CV: coefficient of variation.

### Comparison and analysis of the two prediction models

As shown in Figs 5 and 6, a comparative analysis of the two models was conducted, and the coefficient of determination (R^2^), relative error (RE), and root mean square error (RMSE) were calculated. The model was tested to verify the accuracy of the prediction model, as shown in Table 3.

**Table 3:** Analysis of the estimation accuracy of the water content. The model was selected by the following standard: the larger the R^2^, the smaller the RE and RMSE and the higher the accuracy of the model.

Models	Determination coefficient	Root mean square error	Relative error
	R^2^	RMSE	RE
Random sampling model	0.7413	0.0441	0.0755
Equidistant sampling model	0.8221	0.0365	0.0626

In the stepwise regression model established by different sampling methods, the coefficient of determination of the equidistant sampling model was 0.8221, which was 10.90% higher than that of simple random sampling. The RE and RMSE of the model were relatively high, and the prediction model based on equidistant sampling performed better. The RMSE of 0.0365 and RE of 0.0626 were lower than that of the random sampling model by 17.23% and 17.09%, respectively. These results indicate that the cross variable model based on equidistant sampling is more suitable for predicting the water content of an apple canopy.

## Discussion

This study constructed variables Z_1_ = R_760_ × R_821_, Z_2_ = R_760_ × R_967_, Z_3_ = R_821_ × R_967_, Z_4_ = R_821_ × R_1174_, Z_5_ = R_967_ × R_1174_, and Z_6_ = R_760_ × R_1174_ and predicted the apple canopy water content using the method of systematic sampling. It was found that the prediction accuracy of this model was higher than that established by the simple random sampling method. The results of this study are different from those of previous studies, which may be because the selection of the most sensitive wavelength for different crops is related to the structure of the blade and because differences in leaf structure lead to the different results^1, 4, 7, 12^. Moreover, in the process of data acquisition, the impact of the underlying factors and other factors is expected to lead to different results^21^.

This study shows that the model of apple canopy water content prediction based on equidistant sampling method has better predictive effect than that established by simple random sampling method, because the equidistant sampling method takes into account the the uniform distribution of samples and a sample of each moisture content level was reasonably extracted, Which is consistent with the finding of Jin, Z. X. *et al*. and Brus, D. J., Kempen, B. & Heuvelink G. B. M.^19, 22^, but deferent from the finding of some other scholars^17, 18^. The equidistant sampling method also has certain shortcomings, i.e., the overall number of units may contain hidden forms or “unqualified samples”, and the investigators may neglect them during sample selection. Thus, if we wish to improve the efficiency of sampling, we must have a certain understanding of the overall structure of the sample and make full use of the existing information on the overall unit queue after sampling.

In this paper, stepwise regression analysis is used to analysis the relationship of reflectance of the apple canopy and its water content. Compared to other multiple linear regressions and simple linear analysis, it can automatically select the more important variables in the establishment of the regression equation. Previous studies have shown that the stepwise regression model has a high prediction effect^16, 23^.

In this study, we constructed the cross variables as the independent variable of the prediction model and explored the interactive influence ability between the sensitive wavelengths in the near-infrared band. However, the strong absorption band (middle-infrared band) of leaf water is strongly influenced by the water vapor in the atmosphere. At present, the separation of the contribution of atmospheric water vapor and vegetation water to spectral reflectance features progress, but it still cannot meet the needs of precise quantitative remote sensing in the agricultural field. Thus, further exploration and research are needed^12^.

## Conclusions

A study on the correlation between the tree canopy spectral reflectance and water content, based on the first derivative of the reciprocal logarithm, was presented. The wavelengths of 760 nm, 821 nm, 893 nm, 967 nm, 1114 nm and 1174 nm were selected to construct the cross variables, and the cross variables were Z_1_ = R_760_ × R_821_, Z_2_ = R_760_ × R_967_, Z_3_ = R_821_ × R_967_, Z_4_ = R_821_ × R_1174_, Z_5_ = R_967_ × R_1174_, and Z_6_ = R_760_ × R_1174_. A model was established based on the simple random sampling method, and the significant levels (P < 0.05) of the selected variables were Z_3_, Z_5_, Z_6_; Z_3_ and Z_6_, which reached an extremely significant level (P < 0.01). The stepwise regression model was Y = 0.4797 − 721787.3883 × Z_3_ − 766567.1103 × Z_5_ − 771392.9030 × Z_6_. A model was established based on the equidistant sampling method, and significant levels (P < 0.05) were found for variables Z_2_, Z_5_, and Z_6_; Z_2_ and Z_6_ reached an extremely significant level (P < 0.01). The stepwise regression model was Y = 0.4613 − 480610.4213 × Z_2_ − 552189.0450 × Z_5_ − 1006181.8358 × Z_6_. After verification, equidistant sampling had a superior prediction effect. The calibration set coefficient of determination of 0.6599 and validation set coefficient of determination of 0.8221 were higher than that of the random sampling model by 9.20% and 10.90%, respectively. The RMSE of 0.0365 and RE of 0.0626 were lower than that of the random sampling model by 17.23% and 17.09%, respectively. Therefore, the accuracy of the prediction model of the apple canopy water content was clearly improved when dividing the calibration set and validation set based on the equidistant sampling method. The equidistant sampling method is suitable for predicting the apple canopy water content during the full fruit-bearing period.

## Materials and Methods

### Study area

**Figure 7:**
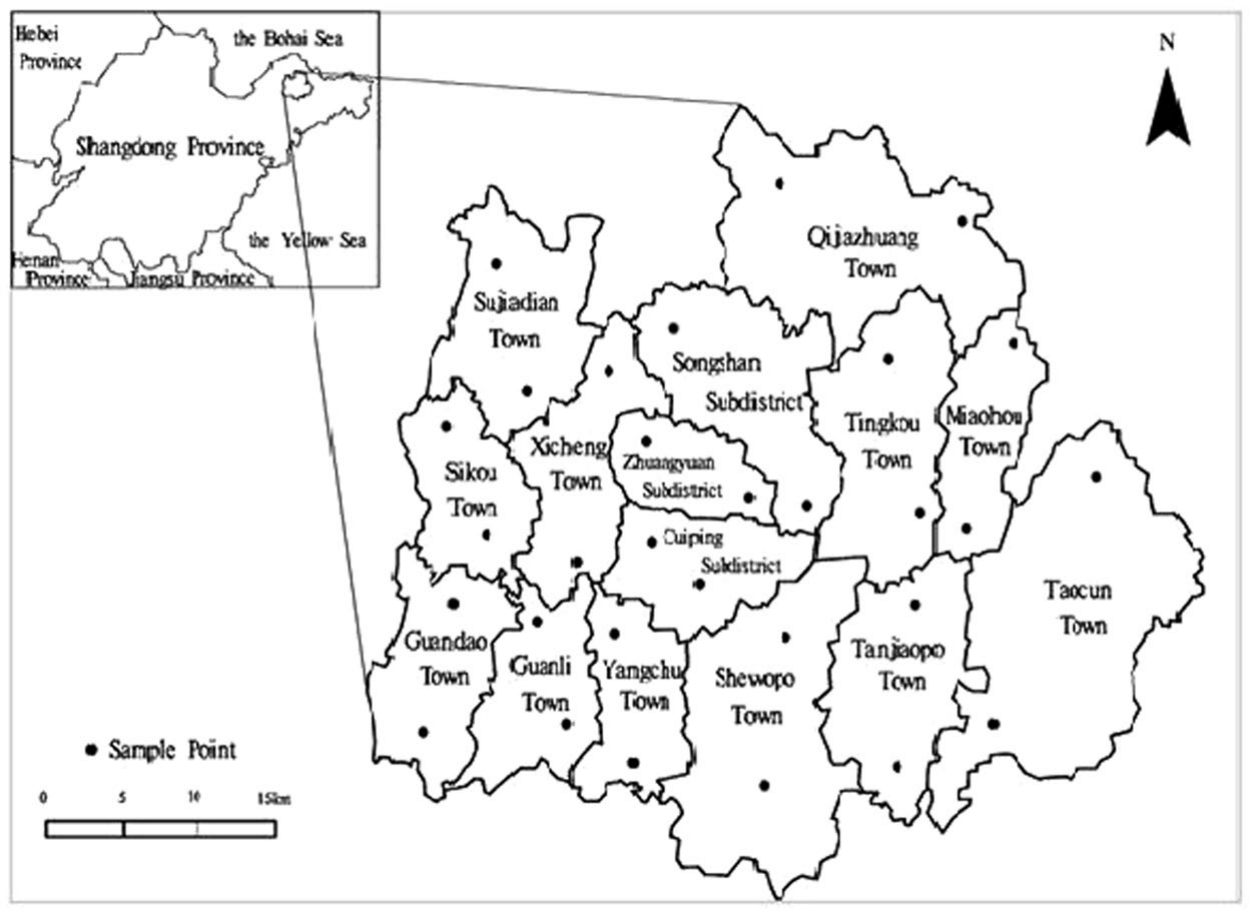
Sampling points of Qixia, Shandong. Thirty orchards were sampled, and the sampling sites were randomly distributed according to the distribution of the orchards. The software used to draw this picture is MapGIS K9. The version number of the software is 1512011184-0060. The software is installed from a CD-ROM without a URL (a URL may be obtained from ZONDY CYBER). First, a project file was built, and a point file and line file were added to the project file. Then, the outline of the map was drawn according to the scale shown in the figure by referring to the map of Shandong province and Qixia City in China. Finally, the specific locations of the sampling points and their names were added to the picture according to the actual distribution of sampling points, and a compass was added.

The apple orchards of Qixia, Yantai City, Shandong Province were selected as the research area. Qixia is located in the central part of the Jiaodong Peninsula (120°33′-121°15′E, 37°05′-37°32′N), which has a mountainous and hilly terrain and a warm temperate monsoon semi-humid climate. The soil is brown soil, with an average annual temperature of 11.3 °C and frost-free period of 207 d. The average annual rainfall is 754 mm. The sampling points are shown in Fig. 7.

### Determination of canopy spectral reflectance

In June 2015, 30 orchards were sampled, and the sampling sites were randomly distributed according to the distribution of the orchards. In each orchard, 3 representative apple trees were selected, and a total of 90 fruit trees were selected. The spectral reflectance of the apple canopy was measured with ASD FieldSpec4, and the band value of the instrument ranged from 350 to 2500. The measurement was conducted in cloudless or partly cloudy weather, and the measurement time was 10:00–15:00. A standard calibration board was used for correction before the measurement. A length of 5 m of fiber was used to measure the canopy to ensure that the entire canopy was in the detection range, reducing the surface reflectance effect on the measurements. The determination process was conducted every 15 min for the correction of a standard board. A standard white board calibration was performed every 15 min.

### Leaf collection and determination of water content

For the simultaneous acquisition of leaf and canopy spectral detection, 12 pieces of healthy and intact leaves were collected around each tree canopy in which the spectral reflectance was measured. The collected leaves were rapidly placed in a bag, which was sealed, numbered, and immediately transferred to a foam ice box and taken back to the lab.

The water content was measured by the drying method. The blade was placed into an envelope bag, and the fresh weight m_1_ was obtained. Then, the bag was placed into an oven at 105 °C for 30 min and then dried at 80 °C to obtain a constant weight, called the dry weight m_2_. According to formula (1), the water content can be calculated as

1 $${\rm{Water}}\,{\rm{content}}=({{\rm{m}}}_{1}-\,{{\rm{m}}}_{2})/{{\rm{m}}}_{1}\times 100 \% $$

### Equidistant sampling method

The total number of samples was arranged in a certain order, and then set a fixed interval according to the sample capacity requirements, and sample extraction was performed from a random starting point according to the fixed interval, which was called equidistant sampling. It is a variant of pure random sampling. During sampling, the ratio of the calibration set and validation set was 4:1 or 3:1. The experimental data of 90 groups were arranged in accordance with the order of water content from low to high, and sample extraction was performed according to the proportion of 4:1, that is, 1 sample was taken at a distance from every 4 samples. The calibration set consisted of 72 groups, and the validation set consisted of 18 groups. The sample statistics are shown in Table 2.

### Random sampling method

The simple random sampling is a sampling method that takes N units as the sample and makes the probability of each sample equal. In this experiment, 18 groups were randomly selected from 90 sets of data as the validation set, and the remaining 72 groups were used as the calibration set. The sample statistics for simple random sampling are shown in Table 1.

### Spectral data processing

Because of the influence of the spectrometer itself and the absorption of atmospheric water, the spectral reflectance contains substantial interference noise. Therefore, before data processing, the error of the spectral data was eliminated, and the 1350–1410 nm, 1820–1940 nm and 2390–2500 nm bands with large noise levels were eliminated.

In the process of spectrum acquisition, the effects of the environment and energy difference of the different bands of the spectrometer response lead to the existence of noise, which comes primarily from the baseline drift and high frequency random noise. Thus, the sample signal is not uniform, i.e., a signal background and light scattering exist, among other nonuniformities. Therefore, it is necessary to smooth the spectral data^23^. The most commonly used smoothing methods include the moving average method, fitting polynomial method, wavelet transform and various regression smoothing methods. Through the comparison of relevant research works^24–27^, the 7-point weighted moving average method is used to smooth the spectral curve in this research. The formula is

2 $${\rm{R}}{^{\prime} }_{{\rm{i}}}=0.15{R}_{{\rm{i}}-3}+0.15{R}_{{\rm{i}}-2}+0.15{{\rm{R}}}_{{\rm{i}}-3}+0.1{{\rm{R}}}_{{\rm{i}}}+0.15{{\rm{R}}}_{{\rm{i}}+1}+0.15{{\rm{R}}}_{{\rm{i}}+2}+0.15{{\rm{R}}}_{{\rm{i}}+3}$$

where R′_i_ is the weighted average value of each of the three points before and after I, and R_i_ is the value of the non-smoothed data points. The weights of the formula are 0.15, 0.15, 0.15, 0.15, 0.15, and 0.15. This approach ensures the smoothness of the spectral curve and guarantees that spectral details are not lost, to lay a suitable foundation for the extraction of sensitive wavelengths.

### Data processing methods

First, the correlations between the canopy water content and the original spectral reflectance, the reciprocal of the original spectral reflectance and the first derivative of the reciprocal logarithm were analyzed. Then, we chose the wavelengths which showed the highest coefficient of determination between the water content and the first derivative of the logarithm of the reciprocal reflectance to construct the cross variables, and the model was established by stepwise regression. Finally, the verification and calibration of the model were partitioned by equidistant sampling, and the model was tested.

R^2^, the RE, and the RMSE were used to test the model and to verify the accuracy of the prediction model. The larger the value of R^2^, the smaller the value of RE and RMSE and the higher the accuracy of the model.

3 $${\rm{RMSE}}=\sqrt{\frac{1}{{\rm{n}}}{\sum }_{{\rm{i}}=1}^{{\rm{n}}}\quad {{(y}_{{\rm{i}}}-{\mathop{{\rm{y}}}\limits^{\wedge }}_{{\rm{i}}})}^{2}}$$

4 $${\rm{RE}}=\frac{1}{n}{\sum }_{{\rm{i}}=1}^{{\rm{n}}}|\frac{{{\rm{y}}}_{{\rm{i}}}-{\mathop{{\rm{y}}}\limits^{\wedge }}_{{\rm{i}}}}{{{\rm{y}}}_{i}}|$$

5 $${{\rm{R}}}^{2}=\frac{{\sum }_{{\rm{i}}=1}^{{\rm{n}}}{({\mathop{{\rm{y}}}\limits^{\wedge }}_{{\rm{i}}}-\overline{{\rm{y}}})}^{2}}{{\sum }_{{\rm{i}}=1}^{{\rm{n}}}{(y}_{{\rm{i}}}-{\overline{y)}}^{2}}$$

where ${{\rm{y}}}_{{\rm{i}}}$ is the measured water content value, ${\hat{{\rm{y}}}}_{{\rm{i}}}$ is the predicted value, $\mathop{{\rm{y}}}\limits^{\bar{} }$ is the average value of ${{\rm{y}}}_{{\rm{i}}}$, and the number of samples is n.

### Stepwise regression analysis

In multivariate regression analysis, if the independent variables are used, a larger sum of squares corresponds to a smaller sum of squared residuals. However, with more variables in the regression equations, the equation of poor stability and the accumulation of interval error in each independent variable will affect the overall error. The reliability prediction of the regression model established in this way is poor and has low accuracy. However, stepwise regression analysis is a method that can automatically select the more important variables in the establishment of the regression equation. Previous studies have shown that the stepwise regression model has a high prediction effect^16, 23^.

The basis is used to introduce and eliminate the variables of stepwise regression analysis.

The following basis is used to introduce a variable:

6 $${{\rm{F}}}_{1i}=\frac{{{\rm{V}}}_{i}({{\rm{x}}}_{1},{{\rm{x}}}_{2},\ldots ,{{\rm{x}}}_{l})}{{{\rm{SS}}}_{s}({{\rm{x}}}_{1},{{\rm{x}}}_{2},\ldots ,{{\rm{x}}}_{l},{{\rm{x}}}_{i})/({\rm{n}}-{\rm{l}}-2)}$$

The critical value ${F}_{\mathrm{in}}$ of *F* test is selected to introduce the independent variables properly. When ${F}_{1i}$ $> {F}_{\mathrm{in}}$, it indicates that the introduction of the independent variable ${{\rm{x}}}_{i}$ is sig nificant; when ${F}_{1i}$ $\le {F}_{\mathrm{in}}$, it indicates that the introduction of the independent variable ${{\rm{x}}}_{i}$ is inconsequential.

The following basis is used to eliminate a variable:

7 $${F}_{2i}=\frac{{{\rm{V}}}_{i}({{\rm{x}}}_{1},{{\rm{x}}}_{2},\ldots ,{{\rm{x}}}_{l})}{{{\rm{SS}}}_{s}({{\rm{x}}}_{1},{{\rm{x}}}_{2},\ldots ,{{\rm{x}}}_{l},{{\rm{x}}}_{i})/({\rm{n}}-{\rm{l}}-1)}$$

The critical value ${F}_{{\rm{e}}}$ of the *F* test is selected to introduce the independent variables properly. When ${F}_{2i}$ $\le \,{F}_{{\rm{e}}}$, it indicates that the introduction of the independent variable ${{\rm{x}}}_{i}$ is inconsequential; when ${F}_{2i}$ $> {F}_{{\rm{e}}}$, it indicates that there are no variables that can be eliminated.

${{\rm{V}}}_{i}$ is the contribution of the independent variable ${x}_{{i}}$ to the sum of squares of regression, n is the sample size, $i$ is the number of arguments that have been introduced, and ${\mathrm{SS}}_{s}$ is the sum of squared residual errors.

The diagnosis of the stepwise regression model must satisfy three conditions: first, the significant levels of P of the variance analysis of equation F must be less than or equal to 0.05; otherwise, the established regression equation cannot be used. Second, the coefficient of the partial correlation coefficient of each regression coefficient is preferably less than or equal to 0.05. Third, residual autocorrelation must exist^28–31^.

### Data availability statement

The experimental data were measured according to the test specifications, which can be used for further analysis.
